# African vaccination week as a vehicle for integrated health service delivery

**DOI:** 10.1186/s12913-015-0989-7

**Published:** 2015-09-02

**Authors:** Richard Mihigo, Blanche Anya, Joseph Okeibunor, Samuel Ajibola, Collins Boakye-Agyemang, Linda Muzenda, Flavienne Issembe, Deo Nshimirimana

**Affiliations:** Immunization, Vaccines & Emergencies, World Health Organization Regional Office for Africa, Cité du Djoué, Brazzaville Republic of Congo; Communication, Advocacy & Media, World Health Organization Regional Office for Africa, Cité du Djoué, Brazzaville Republic of Congo

**Keywords:** Africa, Health, Immunization, Vaccination, Vaccines, Week

## Abstract

**Background:**

African Vaccination Week (AVW) is an initiative of the Member States of the African Region aimed at promoting vaccination and ensuring equity and access to its benefits. The initiative has proven to be particularly effective in reaching populations with limited access to regular health services as well as providing an opportunity to integrate other interventions with immunization services.

**Methods:**

Using data available from the countries within the African Region, the effectiveness of AVW in creating awareness on vaccination as well as providing platform for integrated delivery of other interventions with immunization in the African Region were explored during the 2013 and 2014 campaigns of the AVW.

**Results:**

Countries that participated in the two campaigns of AVW have integrated other interventions with immunization during the AVW. The most common integrated intervention is vitamin A supplementation, followed by deworming. However, other interventions integrated, include public health educational activities, supplementation with vitamins and minerals, provision of other health services as well as introduction of new interventions. In 2013, more than 7,500,000 doses of different vaccine antigens were delivered in17 countries. Vitamin A administered to children under 5 years and women in post-partum in 13 countries with 31,500,000 tablets distributed. Polio eradication campaigns reaching young children in ten countries with 36,711,984 doses of oral polio vaccines (OPV) was the third most common intervention added onto the AVW activities. Over 21,190,000 deworming tablets were distributed to children <5 years and pregnant women in 9 countries. With respect to nutritional interventions, 6,377,222 children were screened for malnutrition in 3 countries while 3,814,680 water, sanitation and hygiene kits were distributed in 3 countries. In 2014, these results were even higher as many more countries integrated multiple interventions in the AVW.

**Conclusion:**

Integration of other interventions with immunization during AVW, in the African Region is common and has shown potentials for improving immunization coverage, as this dedicated period is used both for catch-up campaigns and periodic intensified routine immunization. While its impact may call for further examination, it is a potential platform for integrated delivery of health interventions to people with limited access to regular health service.

## Background

Advances in public health have made significant contribution in improving the health of people. In spite of this, an unacceptably high burden of ill health persists, particularly among the poorest populations, trapping them in a vicious circle of ill health and poverty [1]. This challenge has gained increasing prominence in national, regional and global agenda with growing calls for countries to address it as they strive to attain the Millennium Development Goals. WHO and its partners are engaged in several initiatives to ensure access to vaccination and control vaccine preventable diseases effectively so that millions of people, children, adolescents and adults, may enjoy better health. Experts agree that better access to basic health care in low-income countries depends on better health systems, and this requires innovative solutions to improve access. While a system-wide perspective is recommended, disease-specific initiatives can immediately play a critical role in this effort. To achieve their goals, many of these time-limited initiatives must deliver interventions to all at-risk populations, and must overcome inadequacies in health services in order to do so. One such intervention is immunization of the populations against vaccine preventable diseases to build herd immunity and provision of other supplements in the populations.

Immunization is a proven, cost-effective public-health strategy that has dramatically decreased childhood morbidity and mortality worldwide [2]. Immunization programmes have recorded remarkable successes in the last four decades, contributing to the eradication of smallpox, bringing the eradication of polio within our grasp, controlling of measles and reducing, significantly, the incidence of other vaccine-preventable diseases [3–12]. The Convention on the Rights of the Child and the principles of social justice demand that all children should have equal access to effective childhood vaccines, given the potential of vaccines to save lives [11] and to give every child a chance in life. The effectiveness of school vaccination requirements in preventing disease and introducing new vaccines, in the US as a way of reaching more people, for instance, has been well documented [12–18].

In line with the principles and areas of work outlined in the Global Vaccine Action Plan and under the advice of both the Strategic Advisory Group of Experts (SAGE) on immunization and the Task Force on Immunization (TFI), WHO/AFRO is taking steps to address issues of vaccine preventable diseases by implementing strategies for reaching all eligible persons with effective vaccines. One of the strategies is the implementation of the African Vaccination Week (AVW), which provides a platform for Member States to speak through one collective voice and advocate for immunization as a public health priority in the Region, and achieve high immunization coverage. The overarching objective of the initiative is to target people with limited access to regular health services, thereby working to close the gaps in immunization [19, 20]. The 60^th^ session of the WHO Regional Committee endorsed this initiative with a resolution (AFR/RC60/14), institutionalizing annual AVW for sustaining advocacy, expanding community participation and improving immunization service delivery. This is in tandem with the support of the World Health Assembly for the World Immunization Week contained in its resolutions WHA58.15 and WHA61.15.

WHO/AFRO commenced AVW in 2011 after Member States endorsed it in 2010. The planning for each edition of AVW comes from the countries with inputs from immunization partners, under the coordination of the WHO/AFRO. The countries develop their plans, enumerating the different activities to be undertaken during the week with targets. The plans are shared with the WHO/AFRO. Teleconferences are held to discuss the plan and one planning workshop is held in the WHO/AFRO, Brazzaville to finalize the plans.

The purpose of the AVW celebration is to increase awareness of immunization programmes in the African Region. It creates awareness on the right of every person to be adequately protected against vaccine preventable diseases. The implementation follows definite guidelines. Frequently asked questions (FAQs) as well as reporting templates are also developed to guide the implementers. The country planned activities also give basis for the measurement of outcomes

Typically, the activities undertaken during the week include immunization (catch up campaigns, periodic intensified routine immunization). “Catch up”, here means the conduct of immunization activities to reach those missed with immunization services during the general campaign period. Other activities that get added on include vitamin A supplementation; water, sanitation and hygiene (WASH); deworming, distribution of long lasting insecticide treated bednets, testing for human immunodeficiency virus (HIV) and malaria. The activities target all (infant, children, adolescents, adults, women and elderly) depending on country priorities

Since its first launching in 2011, AVW has grown to become the largest multi-country immunization effort in the Region, with the participation of 43 out of the 47 countries in the Region as at April, 2013. Country participation in AVW was flexible and national authorities chose activities based on their current public health priorities. The potential benefits of integrating other health interventions with immunization are currently on global public health discourse [19]. The Global Immunization Vision and Strategy (GIVS) aptly captured integration as a vital approach to consider in enhancing health for all [21].

As an initiative that primarily aims at increasing public awareness on the benefits of vaccines and immunization, guidelines on integration of other health services were not deliberately enshrined in the AVW systems. Although, it is a yearly event, it can provide a platform to launch integrated health care delivery for the poor and needy. For immunization, it can be an opportunity to deliver additional doses in hard to reach areas. It can deliver antigens for diseases which are endemic such as measles, meningitis, among others. Similarly, for antigens that require multiple doses, it provides an opportunity to identify marginalized populations, create awareness of the antigens among them as well as partner with them to search for defaulters and encourage full immunization.

In the light of these benefits, countries participating in the third and fourth AVW in the African region have taken advantage of this platform to reach the populations with other health interventions during the commemorations. AVW builds on existing child and maternal health days in many countries and thus provides a unique opportunity for delivering other health interventions that focus on access and equity. Activities such as health awareness creation, immunization activities like catch-up with all antigens; periodic intensified routine immunization (PIRI) campaigns and new vaccine introduction lend themselves as opportunities to deliver other health commodities. This paper reviews AVW reports from the participating countries in the African Region to assess the extent to which integration is occurring in these countries as part of the AVW initiative.

## Methods

With the reporting template developed during the planning stage, all participating countries documented the activities they implemented during the week’s celebration. Data was collected on all services delivered and to all populations during the week. The number of doses or quantity of commodities delivered was measured. The level of co-implementation or integration of activities was also gauged. These information were collated as country reports which were submitted to the Regional Office of WHO. The quality of the data was assured with the use of multiple sources of information, such as cross checking of records of supplies and used commodities.

All country reports of activities during the 2013 and 2014 campaigns of the AVW in the African Region were reviewed. Each year, as part of activities for the AVW event, countries were asked to report on achievements of their pre-established AVW vaccination goals, to describe vaccination activities and other integrated public health and training activities undertaken. When applicable, they are encouraged to analyze defined indicators, and to report on resource mobilization, launching events and communication efforts. Forty-three out of 47 countries in the Region submitted their reports. We did not include data from 2011 and 2012 because these were the formative years, when documentation and reporting were not formalized.

Although AVW is officially a one week event, most countries extend their vaccination activities, particularly campaigns, over the course of several weeks. As a reference the AVW vaccination and communication activities were consolidated into broad categories and listed by countries (see Tables 1 and 2). To summarize the integration of other preventive health interventions during the AVW, all other interventions implemented were identified and countries grouped by intervention, listing target populations.

**Table 1:** Main activities conducted in 2013 and 2014 AVWs by countries

Interventions planned/conducted during African Vaccination Week (AVW)	African Vaccination Week (AVW) 2013		African Vaccination Week (AVW) 2014	
	Number of countries	List of countries	Number of countries	List of countries
Communication activities			47	
• Advocacy, sensitization, social mobilization, production of information, education and communication (IEC) materials, training/media briefing				All 47 countries in the African Region
				Mauritius & Uganda
				Kenya
• Short message services (SMS) campaign				
• Award to districts with good performance in immunization				
Polio campaign	10	Benin, Burkina Faso, Cameroon, Chad, Côte d’Ivoire, Guinea, Liberia, Mali, Mozambique, Sierra Leone	7	Equatorial Guinea, Mali, Gambia, Niger, Burkina Faso, Benin, Central African Republic
Catch-up vaccination activities	17	Angola, Algeria, Benin, Burkina Faso, Chad, Congo, Cameroon, DRC, Gabon, Guinea, Kenya, Lesotho, Madagascar, Mauritania, Swaziland, Togo, Zimbabwe	32	Angola, Algeria, Benin, Botswana, Burkina Faso, Burundi, Cap Verde, Chad, Côte d’Ivoire, Central African Republic, Congo, Comoros, DRC, Ethiopia, Eritrea, Gabon, Ghana, Guinea, Guinea Bissau, Lesotho, Malawi, Madagascar, Mozambique, Namibia, Nigeria, Sao Tome, Senegal, Togo, Swaziland, Uganda, Zambia, Zimbabwe
Vitamin A administration	13	Angola, Benin, Cameroon, Congo, Côte d’Ivoire, Gabon, Guinea, Liberia, Madagascar, Mozambique, Rwanda, Swaziland, Nigeria	20	Angola, Botswana, Cameroon, Comoros, Eritrea, Gambia, Ghana, Guinea, Liberia, Madagascar, Mauritania, Mozambique, Nigeria, Namibia, Säo Tomé, South Sudan, Swaziland, Rwanda, Zambia, Togo
Deworming tablets	9	Cameroon, Côte d’Ivoire, Gabon, Guinea, Liberia, Madagascar, Mozambique, Nigeria, Zimbabwe	15	Angola, Botswana, Cameroon, Comoros, Gambia, Guinea, Liberia, Madagascar, Mauritania, Mozambique, Nigeria, Rwanda, Swaziland, Zambia, Togo
Malnutrition screening	3	Cameroon, Madagascar, Mozambique	5	Chad, Ghana, Mauritania, Madagascar, Rwanda
New vaccine introduction			7	Angola and Congo;
• Rotavirus				Lesotho and Swaziland;
• Pneumococcal conjugate Vaccine (PCV)				Rwanda, Tanzania and Seychelles.
• Human Papillomavirus Vaccine (HPV)				
• Measles 2^nd^ dose				Tanzania
Combined with Child Health days			6	Botswana, Cameroon, Ghana, Madagascar, Nigeria, Rwanda
Distribution of Long Lasting Insecticide Treated Bednets (LLITNs)			4	Angola, Chad, Congo, Guinea Bissau

### Ethical considerations

This paper is based on routine programme activities and not typical research. The data from the activities were considered useful for advancement of programming in the area of health services delivery. Since it was not a typical study, no ethical approval was sought. All the same, the World Health Organization, as an organization with an established Ethical Review Committee is conversant with the rules and respects them.

## Results

The 2013 and 2014 AVW were the third and fourth to be celebrated in the African Region. Each showed great improvement over the previous years in terms of number of countries involved and the delivery of services. In 2013, all countries except Central Africa Republic (CAR), Equatorial Guinea and Namibia participated in the third AVW devoted to the theme “Save lives, prevent disabilities, vaccinate!” between 22 and 28 April, 2013 and coincided with the 2nd World Immunization Week (WIW). Participating countries also embarked on mobilization and sensitization campaigns, using traditional, modern and social media, engaging religious leaders, organizing sensitization workshops for media practitioners and health workers, among others; conducting community dialogues through panel discussions, recognizing deserving health workers through award of certificates, and sensitizing supervisors as well as undertaking supportive supervisory visits to vaccination sites. These activities had a common overarching goal: to show the benefits of vaccination in promoting public health.

In addition to this, the countries used the AVW to introduce some new vaccines like the pneumococcal conjugate vaccine, rotavirus and human papillomavirus (HPV) vaccines, provided vitamin A supplementation and deworming medicines for intestinal worms, distributed Long-Lasting Insecticide-Treated Nets, provided traditional vaccines as part of ‘catch up’ activities in low performing districts, conducted polio and measles campaigns, screened children for moderate or severe malnutrition, distributed water sanitation and hygiene (WASH) kits, oral rehydration salt (ORS), iron and folate tablets as well as condoms. These activities served several purposes: raising awareness on the life-saving value of immunization; reaching underserved and marginalized communities (particularly those living in remote areas, deprived urban settings and strife-torn areas) with high-impact child survival packages; reinforcing the medium and long-term benefits of immunization and other child survival interventions, all with the aim of increasing vaccination coverage and helping to transform the lives of millions of children, by giving them a chance to grow up healthy, go to school, and improve their life prospects.

A wide range of vaccination activities were implemented under the umbrella of AVW. These include intensification of the routine programme to complete vaccination schedules: polio eradication campaigns, catch-up vaccination activities. Other interventions include Vitamin A administration, deworming and nutritional interventions (Table 1). More than half (55.8 %) of the 43 participating countries integrated other interventions into the AVW activities for 2013. Interventions integrated, however, varied in number and type. The number ranged from one in Algeria, DRC, Kenya, Lesotho, Mali, Mauritania, Rwanda and Togo, to five in Cameroon. In-between, Angola, Burkina Faso, Chad, Congo, Nigeria, Swaziland and Zimbabwe integrated two interventions, while Benin, Côte d’Ivoire, Gabon and Liberia integrated three interventions. Another set of countries integrated four interventions. These included Guinea, Madagascar and Mozambique.

At this point, it may be important to reiterate that the main goal of the AVW in the African Region is to create awareness on the importance of immunization against vaccine preventable diseases, and to get the governments and communities to appreciate their rights and responsibilities in ensuring that everybody gets adequately immunized. In addition to this, actual immunization activities as well as other health interventions were undertaken. The commonest intervention integrated with AVW activities of awareness creation, were catch-up vaccination activities in 17 countries with more than 7,500,000 doses of different antigens administered. This was followed by Vitamin A administration to children under 5 years and women in post-partum in 13 countries with 31,500,000 tablets distributed. Polio eradication campaigns reaching young children in ten countries with 36,711,984 doses of oral polio vaccines (OPV) was the third most common intervention added onto the AVW activities in 2013. Over 21,190,000 deworming tablets were distributed to children <5 years and pregnant women in 9 countries. With respect to nutritional interventions, 6,377,222 children were screened for malnutrition in 3 countries while 3,814,680 WASH kits were distributed in 3 countries.

In 2014, many more countries delivered multiple interventions along the primary goals of creating awareness on immunization. This year, the AVW was covered by the news media in all 47 countries of the Region, including South Africa that did not celebrate the event. The event was also promoted through the official WHO website and twitter account, news agencies, newspapers/magazines, radio/TV, online and other media.

The twitter campaign was held from 17 March 2014 till 24 April 2014. A fourteen percent (14 %) increase in twitter followers was achieved during the period. Tweets contained links to documents, images, videos and stories for more information. Topics covered include AVW announcement; background information; past success of AVW; planned activities for AVW 2014; regional and country launch of AVW; vaccine safety; importance of vaccination; other interventions during AVW; vaccination of adults; vaccination in crisis situations; pneumonia and diarrheal diseases; human papillomavirus (HPV), as well as cervical cancer, measles, and polio. In all the participating countries, the President or representative of the President (Minister of Health or wife of the President) launched the campaign in 2014.

Fifteen of the 47 countries in the African Region celebrated the 2014 edition of the African Vaccination Week (AVW), with the theme “Vaccination: a shared responsibility” within the week of 22 to 27 April 2014. The celebration of the event however continued in the remaining 32 countries in the Region in the months of May and June 2014. Reports from Member States indicate that countries used the occasion to hold round-table discussions, advocacy and social mobilization activities for immunization, training sessions, introduction of new vaccines into national routine immunization programmes; provision of life-saving interventions such as deworming, vitamin A supplementation, distribution of mosquito nets, growth monitoring. Others include ‘catch up’ vaccination activities against diseases such as polio, measles, diphtheria, whooping cough, neonatal tetanus, influenza, yellow fever, rotavirus, bacterial pneumonia. See Table 1 for details.

Further, in 2014, many more interventions were brought on board, in addition to the interventions that were delivered in 2013. The other interventions include the introduction of new vaccines. Countries like Angola and Congo took advantage of the AVW 2014 to introduce rotavirus vaccine while Lesotho and Swaziland introduced PCV. Rwanda, Lesotho, South Africa and Seychelles introduced HPV vaccines while Tanzania introduced the second dose of measles vaccine.

**Fig. 1:**
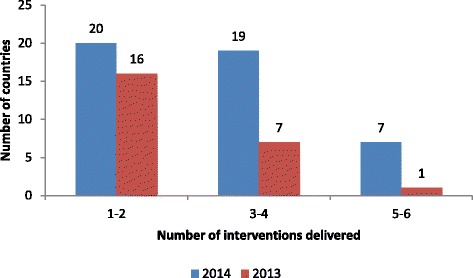
Number of interventions delivered during African Vaccination Week (AVW) by number of countries and year of AVW

Figure 1 further elaborates on the comparative improvement recorded in 2014. It shows that while only one country added up to five interventions to the conventional AVW activities in 2013, as many as 7 countries made similar addition in 2014. Nineteen countries delivered between 3 and 4 interventions in 2014 compared to seven in 2013. Similarly, 20 countries added at least one other intervention in 2014 compared to 16 in 2013.

## Discussion

AVW provides a veritable platform for an integrated and comprehensive public health service targeting hard to reach groups along with introduction of new vaccines to contribute towards efforts to attain the health MDGs. Despite the fact that the integration of other preventive interventions did not form part of the original design for the AVW initiative at the Regional level, lessons learnt from the 2013 and 2014 AVW indicate that it is a viable option. Within the flexible AVW framework, countries took advantage of its platform to implement other interventions successfully, thus addressing some health conditions that make realization of the health MDGs seem like a mirage. The increase in number of countries and commodities delivered during the week is a proof of the potential of the AVW for delivering multiple interventions. It shows the appreciation of the feasibility of driving the integration goals on the AVWs. This in itself is a success story for the AVW.

Although coverage of childhood immunization is relatively high, much more needs to be done to save children from other preventable causes. Every opportunity must be used to increase coverage of effective interventions and prevent child deaths.

Although it is evident that AVW campaigns have contributed immensely towards tackling vaccine preventable deaths, it is clear that there is no single approach that fully addresses the problem of pneumonia and diarrhoea. It requires protecting children by providing a healthy environment, preventing children from becoming ill and treating them when they become ill. WHO, UNICEF and partners have developed the global action plan to prevent and control pneumonia and diarrhoea. There is uniform consensus on which interventions are effective. These could be integrated easily into the AVW. AVW builds upon the core principles of primary health care, namely universal access to care and coverage; commitment to health equity, community participation and inter-sectoral approach to health care [22]. It creates health awareness on vaccine preventable diseases and immunization services in communities previously unreached by the routine health service, thereby covering more people and giving them access. This is an effective and efficient model for integrated delivery of appropriate health interventions at the community level in Africa [23]. The integrated delivery of interventions during the AVW celebration proved feasible where adequate supplies of commodities and other intervention materials were made available. Communities, health workers, policy makers and other stake holders were supportive. Their buy-in in a more formalized process of integrated delivery of intervention using AVW will prove a worthy investment over time. The envisaged constraints are mainly due to social factors, namely acceptability for a given intervention, health system issues like availability of interventions as well as national health policies about provision of certain interventions like anti-malarials. Children could be protected by 6 actions, of which exclusive breastfeeding for 6 months is an important one. Pneumonia and diarrhoea could be prevented by vaccination. Children with pneumonia and diarrhoea could be treated by standard case management with low cost but effective antibiotics, ORS and Zinc at community level, health centres and hospitals. The AVW provides platform to educate mothers on these and prevent childhood mortalities linked to these problems.

In addition to vaccination, vitamin A and deworming during immunization sessions can include health talk on malaria prevention; improve care seeking, case management of childhood illness and provision of Oral Rehydration Salts to children with diarrhoea. Other interventions could include identification of human immunodeficiency virus (HIV) exposed infants, adolescents; growth monitoring and nutrition counseling where needed; hand washing demonstrations and soap distribution if available; chlorine promotion and distribution if available and HPV vaccine for adolescent girls as well as HIV prevention and testing

### Limitation

This paper is based on reports and programme records. Though verifiable and actually verified for validity and reliability, the paper lacks the benefits of deliberately designed survey to interact with recipients. The lesson from this, as the programme moves to the 5^th^ edition is to plan a survey with more robust analysis.

## Conclusion

This paper provides insight into the level and nature of integration that could occur during AVW and suggests that African countries are increasingly and successfully integrating other interventions with immunization. In 2014 more countries integrated interventions during AVW efforts after media campaigns supported by WHO regional and sub-regional offices. This summary of integration during AVW points to the need to promote better and more complete reporting of integrated activities and may serve as a baseline to plan for additional evaluations of integration practices in the African Region in the future.

## Abbreviations

AFR: African Region
AVW: African Vaccination Week
CAR: Central African Republic
DRC: Democratic Republic of Congo
GIVS: Global Immunization Vision and Strategy
HIV: Human Immunodeficiency Virus
HPV: Human Papillomavirus
IEC: Information Education and Communication
LLITN: Long Lasting Insecticide Treated Nets
MDG: Millennium Development Goal
ORS: Oral Rehydration Salt
PCV: Pneumococcal Conjugate Vaccine
SAGE: Strategic Advisory Group of Experts
SMS: Short Message Service
TFI: Taskforce on Immunization
UNICEF: United Nations Children Fund
WASH: Water Sanitation and Hygiene
WHA: World Health Assembly
WHO: World Health Organization
WHO/AFRO: WHO Regional Office for Africa
WIW: World Immunization Week
